# Small-angle X-ray scattering data of a guanine-rich DNA derived from the promoter region of c-MYC gene in solution

**DOI:** 10.1016/j.dib.2022.108285

**Published:** 2022-05-17

**Authors:** Kohei Miyauchi, Hiroshi Imamura, Yudai Yamaoki, Minoru Kato

**Affiliations:** a Graduate School of Life Sciences, Ritsumeikan University, 1-1-1 Nojihigashi, Kusatsu, Shiga 525-8577, Japan; b Department of Applied Chemistry, College of Life Sciences, Ritsumeikan University, 1-1-1 Nojihigashi, Kusatsu, Shiga 525-8577, Japan; c Institute of Advanced Energy, Kyoto University, Gokasho, Uji, Kyoto 611-0011, Japan

**Keywords:** c-MYC, DNA, G-quadruplex, SAXS, Solution structure

## Abstract

This article presented the small-angle X-ray scattering (SAXS) data of a guanine-rich DNA derived from the promoter region of c-MYC gene (Pu22) in solution. The data is collected under the condition, where the Pu22 takes a guanine quadruplex (GQ) structure. The SAXS curve was also measured and analyzed when 18-crown-6, a chelator of K^+^ ions, was added to the Pu22 solution.

## Specifications Table

**Table:** 

Subject	Structural Biology
Specific subject area	Small-angle X-ray scattering analysis
Type of data	Figure (SAXS curves represented as log-log, Krakty, and Guinier plots)
How the data were acquired	Small-angle X-ray scattering data of the synthesized DNA oligonucleotide of c-MYC gene (Pu22) were collected on a BL-10C beamline at the Photon Factory (PF) of the High Energy Accelerator Research Organization (KEK) in Tsukuba, Japan. The single experiment was conducted within several hours.
Data format	Raw, Analyzed
Description of data collection	DNA stock solution was prepared at the laboratory in Ritsumeikan University and shipped at 4 °C to the beamline. The sample was prepared at the beamline and irradiated. 2D images (TIFF format) were collected and circularly averaged (1D data). Data shown is buffer-subtracted SAXS curve.
Data source location	Ritsumeikan University, Kusatsu, Japan
Data accessibility	Repository name: Small Angle Scattering Biological Data Bank (SASBDB) Data identification number: SASDNK7 and SASDNL7 Direct URL to data: https://www.sasbdb.org/data/SASDNK7 https://www.sasbdb.org/data/SASDNL7

## Value of the Data

• The present small-angle X-ray scattering (SAXS) data can be used to analyze solution structures of guanine quadruplexes (GQ), which are four-stranded secondary structures of DNA and play key roles in the regulation of replication and transcription.
• The presented SAXS data can benefit the researchers who conduct experiments and computational modeling for understanding the mechanism of GQ formation in solution.
• The SAXS data in the presence or absence of K^+^ ions, which stabilize the structure of GQ, will be useful to reveal the effect of K^+^ ions on the global structure of GQ.
• The SAXS data may be used for integrative structural modeling, where the SAXS, spectroscopic (e.g., circular dichroism), and molecular dynamics simulation data are combined.

## 1 Data Description

**Fig. 1:**
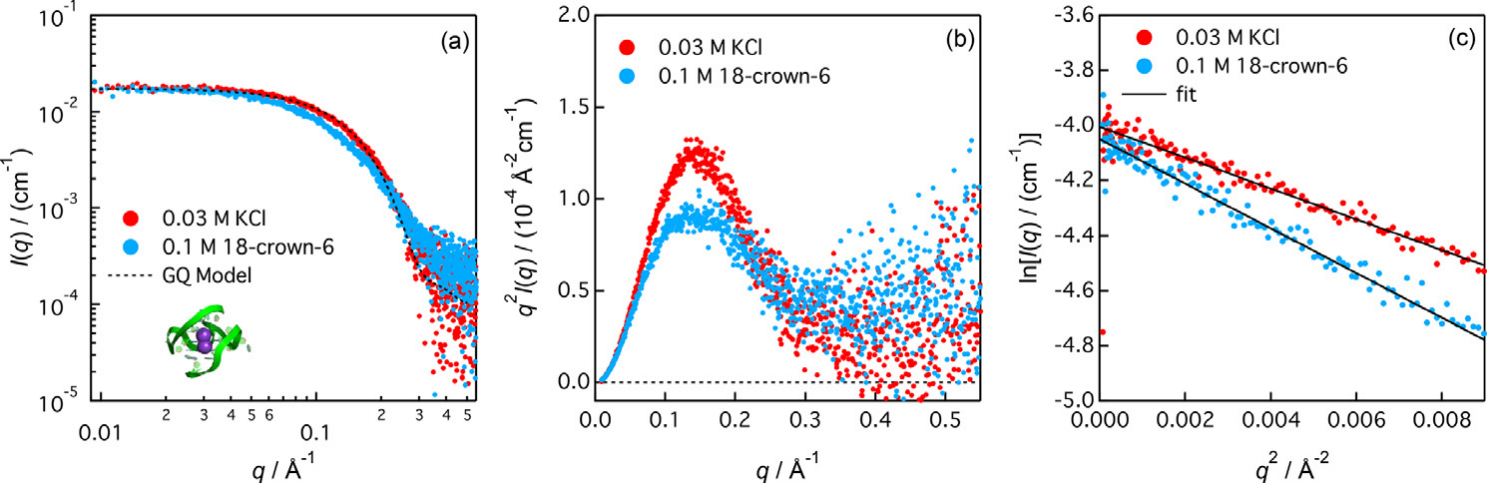
Small-angle X-ray scattering (SAXS) of Pu22 in 0.05 M Tris-HCl solution (pH 8.0) with 0.03 M KCl or 0.1 M 18-crown-6. SAXS curves are presented as (a) log-log, (b) Kratky, and (c) Guinier plots. The black dotted line in (a) indicates the theoretical SAXS curve of the GQ model of Pu22 (PDB: 1XAV; purple spheres represent K^+^ ions). Solid lines in (c) indicate Guinier approximation (Eq. (2)).

A guanine-rich DNA is derived from the promoter region of c-MYC gene that predominantly forms a parallel-type GQ structure [1] and it is an attractive drug target for cancer therapy. Here, we used a mutated sequence (Pu22: 5′-d(TGAGGGTGGGTAGGGTGGGTAA)-3′) that reportedly forms the same parallel-type GQ structure, exclusively [1]. Fig. 1 shows the SAXS curves of Pu22. The SAXS curve of Pu22 with 0.03 M KCl (red circles, Fig. 1a) is consistent with the theoretical scattering curve calculated based on its GQ structure of Pu22 (black dotted line, Fig. 1a) using a CRYSOL program [2], where the default parameters were used and a fitting was applied. The log-log (Fig. 1a) and Kratky plots (Fig. 1b) of Pu22 with 0.03 M KCl are distinct from that with 0.1 M 18-crown-6 (1,4,7,10,13,16-hexaoxacyclooctadecane), which was added to remove K^+^ ions from the GQ structure of Pu22. Guinier analysis in Fig. 1c gave the radii of gyration (*R*_g_); 13.0 ± 0.1 Å (with 0.03 M KCl) and 15.6 ± 0.1 Å (with 0.1 M 18-crown-6).

## 2 Experimental Design, Materials and Methods

### 2.1 Sample

The sequence of Pu22 [1] is 5′-d(TGAGGGTGGGTAGGGTGGGTAA)-3′, also referred to as MYC22-G14T/G23T [1]. The synthesized Pu22 was purchased from FASMAC Co., Ltd. (Kanagawa, Japan). The molecular weight is 6991.6, and the extinct coefficient at 260 nm is 2.287 × 10^5^ L mol^−1^ cm^−1^. The lyophilized powder of Pu22 was dissolved at ∼5 mg/mL and dialyzed at 4 °C with a buffer solution containing 0.05 M Tris-HCl (pH 8.0) and 0.03 M KCl or a buffer solution containing 0.05 M Tris-HCl (pH 8.0) and 0.1 M 18-crown-6. A membrane with a cutoff of 3.5 kDa (Scienova GmbH, Germany) was used for the dialysis. The ultraviolet absorption was measured by V-630Bio (JASCO Co., Ltd., Japan) using quartz windows with a 0.1 cm path length to determine the concentration. The dialyzed DNA solutions were diluted to 1.8 mg/mL for Pu22 with 0.03 M KCl and 1.5 mg/mL for Pu22 with 0.1 M 18-crown-6. Before the SAXS measurement, the sample solution was heated at 95 °C for 10 min and cooled down to 25 °C.

### 2.2 Small-Angle X-ray Scattering (SAXS) Measurements

The SAXS experiments were performed with a BL-10C beam line at the Photon Factory (PF) of the High Energy Accelerator Research Organization (KEK) in Tsukuba, Japan [3]. The X-ray wavelength (*λ*) was 0.15 nm. The camera length was 1 m, which was calibrated based on the scattering pattern of silver behenate [4]. The X-ray intensities were acquired by a PILATUS3 2M detector (DECTRIS Ltd., Switzerland). The temperature was controlled to be at 25 ± 0.1 °C. The sample was placed in a cell with 50 μm-thick quartz windows. The exposure time was 60 s (2 s/image). The buffer solutions were also measured as backgrounds. The circular 1D averaging of the images and the background subtraction was performed using SAngler version 2.1.58 [5]. The 1D scattering intensity data were averaged. The scattering parameter *q* is defined as *q* = |***q***| = 4πsin*θ*/*λ*, where ***q*** is the scattering vector, and 2*θ* is the scattering angle of the X-rays. The *q*-range available was 0.0086 < *q* < 0.565 Å^−1^. The absolute scattering intensity of the DNA (*I*(*q*)) (cm^–1^) was determined by:

(1) $$I\left(q\right)=\left[\left(\frac{I_{\text{Samp}\&\text{Cell}}}{T_{\text{Samp}\&\text{Cell}}}-\frac{I_{\text{Cell}}}{T_{\text{Cell}}}\right)-\phi \left(\frac{I_{\text{Back}\&\text{Cell}}}{T_{\text{Back}\&\text{Cell}}}-\frac{I_{\text{Cell}}}{T_{\text{Cell}}}\right)\right]/CF,$$

where *I* and *T* are the scattering intensity per unit time and the transmittance, respectively. The subscripts Cell, Back&Cell, and Samp&Cell indicate the data using a cell, solvent and cell, and solution and cell, respectively. *ϕ* is 1 – (*cν*/1000), where *c* is the DNA concentration (mg/cm^3^) and *ν* is the partial specific volume of DNA (0.57 g/cm^3^) [6]. *CF* is the correction factor to convert the observed intensity in arbitrary units into the absolute intensity. The *CF* value depends on the experimental setup and was determined by water scattering [7] as a standard.

The scattering in the low-*q* region is described by:

(2) $$lnI\left(q\right)=-\frac{1}{3}{R_{g}}^{2}q^{2}+lnI\left(0\right),$$

where *R*_g_ is a radius of gyration according to the Guinier law [8]. The *q* regions for the fitting were estimated using the program AUTORG [9] in the ATSAS 2.8.3 software [10]; *q*_max_*R*_g_ was less than 1.3, where *q*_max_ is the largest *q* for the regression. A theoretical scattering profile of GQ structure of Pu22 (PDB: 1XAV) was calculated using the atomic coordinates with the CRYSOL program [2].

The scattering data were deposited in the Small Angle Scattering Biological Data Bank (SASBDB) [11] under the following accession codes: SASDNK7 and SASDNL7. The columns in the raw data (.dat format) were listed the order: scattering parameter (*q* / Å^–1^), the intensities (*I*(*q*) / cm^–1^) and errors on the intensities (σ(*I*(*q*)) / cm^–1^), i.e.,:

$$\begin{array}{ccc} \text{COLUMN}\;1 & \text{COLUMN}\;2 & \text{COLUMN}\;3\\ q & I\left(q\right) & \sigma \left(I\left(q\right)\right) \end{array}$$

The log-log plot in Fig. 1(a) was generated by taking the base-10 logarithm of *q* and *I*(*q*), i.e., log*I*(*q*) vs. log*q*. The plots *q*^2^*I*(*q*) vs. *q* and ln*I*(*q*) vs. *q*^2^ are the Kratky plot in Fig. 1(b) and the Guinier plot in Fig. 1(c), respectively. The .dat file recorded some comments and logs during SAXS profile generation indicated by a hash mark (#).

## Ethics Statements

None.

## CRediT authorship contribution statement

**Kohei Miyauchi:** Conceptualization, Investigation, Writing – review & editing. **Hiroshi Imamura:** Conceptualization, Investigation, Writing – original draft, Writing – review & editing. **Yudai Yamaoki:** Writing – review & editing. **Minoru Kato:** Supervision.

## Declaration of Competing Interest

The authors declare that they have no known competing financial interests or personal relationships that could have appeared to influence the work reported in this paper.

## Data Availability

Guanine-rich DNA derived from the promoter region of c-MYC gene (MYC22-G14T/G23T) with KCl (Original data) (SASBDB: SASDNK7) and Guanine-rich DNA derived from the promoter region of c-MYC gene (MYC22-G14T/G23T) with 18-crown-6 (Original data) (SASBDB: SASDNL7).
